# The Application of Low-Frequency Transition in the Assessment of the Second-Order Zeeman Frequency Shift

**DOI:** 10.3390/s21248333

**Published:** 2021-12-13

**Authors:** Yang Bai, Xinliang Wang, Junru Shi, Fan Yang, Jun Ruan, Ruifang Dong, Shougang Zhang

**Affiliations:** 1 National Time Service Center, Chinese Academy of Sciences, Shu Yuan Road, Xi’an 710600, China; baiyang@ntsc.ac.cn (Y.B.); shijunru@ntsc.ac.cn (J.S.); yangfan161@126.com (F.Y.); ruanjun@ntsc.ac.cn (J.R.); dongruifang@ntsc.ac.cn (R.D.); szhang@ntsc.ac.cn (S.Z.); 2 Key Laboratory of Time and Frequency Primary Standards, National Time Service Center, Chinese Academy of Sciences, Xi’an 710600, China; 3 University of Chinese Academy of Sciences, Yu Quan Road, Beijing 100049, China

**Keywords:** cesium atomic fountain clock, second-order Zeeman frequency shift, low-frequency transition

## Abstract

Second-order Zeeman frequency shift is one of the major systematic factors affecting the frequency uncertainty performance of cesium atomic fountain clock. Second-order Zeeman frequency shift is calculated by experimentally measuring the central frequency of the (1,1) or (−1,−1) magnetically sensitive Ramsey transition. The low-frequency transition method can be used to measure the magnetic field strength and to predict the central fringe of (1,1) or (−1,−1) magnetically sensitive Ramsey transition. In this paper, we deduce the formula for magnetic field measurement using the low-frequency transition method and measured the magnetic field distribution of 4 cm inside the Ramsey cavity and 32 cm along the flight region experimentally. The result shows that the magnetic field fluctuation is less than 1 nT. The influence of low-frequency pulse signal duration on the accuracy of magnetic field measurement is studied and the optimal low-frequency pulse signal duration is determined. The central fringe of (−1,−1) magnetically sensitive Ramsey transition can be predicted by using a numerical integrating of the magnetic field “map”. Comparing the predicted central fringe with that identified by Ramsey method, the frequency difference between these two is, at most, a fringe width of 0.3. We apply the experimentally measured central frequency of the (−1,−1) Ramsey transition to the Breit-Rabi formula, and the second-order Zeeman frequency shift is calculated as 131.03 × 10^−15^, with the uncertainty of 0.10 × 10^−15^.

## 1. Introduction

Recently, atomic clocks have played an important role in the field of basic research and engineering technology [1,2,3], as the reference clock [4,5,6,7,8], cesium atomic fountain clock, calibrates other atomic clocks with the highest accuracy. There are several factors that limit the development of frequency accuracy [9,10,11,12,13,14]. The major frequency correction is the second-order Zeeman frequency shift [15], which is caused by the static magnetic field, traditionally called the C-field [16], which is applied in the region extending from below the Ramsey microwave cavity to well above the top of the atomic parabolic flight. Second-order Zeeman frequency shift and its uncertainty are calculated by measuring the central frequency of the (1,1) or ((−1,−1)) magnetically sensitive Ramsey transition fringes in space and time domains. Additionally, the measurement and adjustment of the C-field is crucial for the correction of second-order Zeeman frequency shift. However, subjected to the cut-off waveguide of the Ramsey cavity, there exists a certain dead zone (approximately 6 cm) in terms of acquiring the magnetic field distribution by microwave transition [17,18,19,20]. By way of replacement, the magnetic field is measured by applying a low-frequency magnetic field coil, in order to excite the Δ*F* = 0, Δ*m_F_* ≠ 0 magnetically sensitive transition; this is called the low-frequency transition method [16,21]. The central frequency of the low-frequency resonance curve can be identified in order to calculate the magnetic field at a particular apogee. Additionally, the low-frequency transition method is not affected by the uniformity of the C-field, and the low-frequency electromagnetic waves can enter the Ramsey cavity and cut-off waveguide, which can realize the measurement of the magnetic field inside the Ramsey cavity. Therefore, it is an optimal method to measure the magnetic field of the cesium atomic fountain clocks. The central fringe of the (−1,−1) magnetically sensitive Ramsey transition can be predicted by a numerical integration of the magnetic field “map”, and the difference between the predicted fringe and the measured fringe is less than 0.3 in width. Accordingly, the magnetic field distribution obtained by the low-frequency transition method can determine the position of the central fringe of the (−1,−1) magnetically sensitive Ramsey transition, and then the second-order Zeeman frequency shift can be calculated.

## 2. Theoretical Study

**Figure 1:**
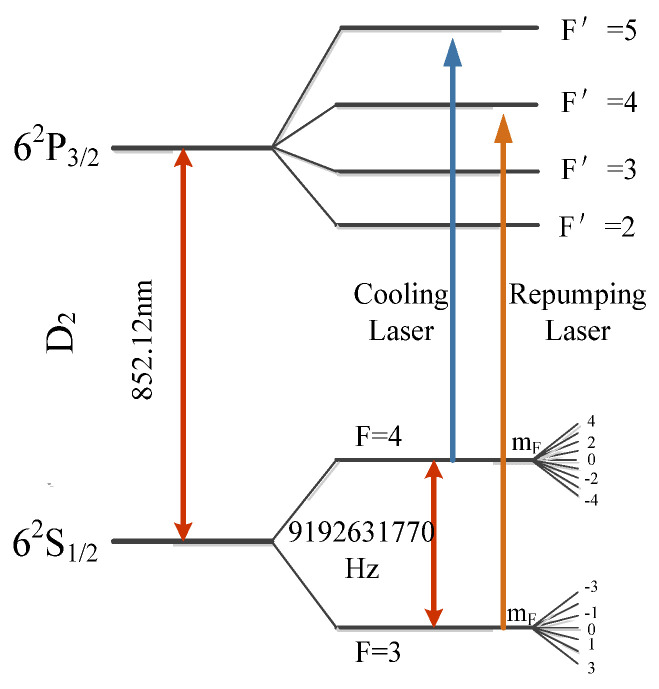
Hyperfine levels of ^133^Cs.

Figure 1 shows the transitions between the ground state and the excited state of the cesium atom, which contains three kinds of transition band. Cesium atom D_2_ line 6^2^S_1/2_*F* = 4→6^2^P_3/2_*F*’ = 5 and 6^2^S_1/2_*F* = 3→6^2^P_3/2_*F*’ = 4 transitions in the light frequency band, are used for trapping, cooling, launching, and detecting the atoms. The transition of |*F* = 3〉 →|*F* = 4〉 corresponding to the microwave frequency band is used for the excitation and state-selection of atoms and the measurement of the C-field along the atomic flight trajectory [17,18,19,20]. The cesium atom ground-state splits into 2*F* + 1 Zeeman sublevels when there is an external magnetic field, and the transition between Zeeman sublevels can be used to measure the magnetic field distribution by the low-frequency transition method [16,21].

The Breit-Rabi formula [22] gives the energy of cesium atom’s ground-state hyperfine transition in the C-field:

(1) $$E\left(F,m_{F}\right)=-\frac{E_{HFS}}{2\left(2I+1\right)}-g_{I}\mu_{B}B_{0}m_{F}\pm \frac{1}{2}E_{HFS}{\left(1+\frac{4m_{F}}{2I+1}x+x^{2}\right)}^{1/2}$$

where *E_HFS_* = *h*ν*_Cs_*, is the photon energy corresponding to the unperturbed cesium atom ground-state hyperfine transition, ν*_Cs_* = 9,192,631,770 Hz is the unperturbed cesium atom ground-state hyperfine transition frequency, and *h* is Planck’s constant. *x* = (*g_J_* + *g_I_*)*μ*_*B*_*B*_0_/*E_HFS_*, for the magnetic field strength used in the cesium atomic fountain clock, *x* << 1, *g*_*J*_ and *g_I_* are the electronic and nuclear *g* factors, *g_J_* = 2.002540 and *g_I_* = 0.4013 × 10^−3^, respectively, *μ_B_* is Bohr’s magneton, *m_F_* is magnetic quantum number, and *I* is the nuclear angular momentum. In this equation, the minus sign always applies in front the square root when *F* = *I* − *S*. When *F* = *I* + *S*, the plus sign applies. 

To approximately expand the Breit-Rabi formula, the transition frequency between different hyperfine Zeeman sublevels is:

(2) $$\nu_{\left(F=3,m_{F}\right)-\left(F=4,m_{F}\right)}=\nu_{Cs}\left[1+\frac{1}{4}m_{F}x+\frac{1}{2}\left(1-\frac{1}{16}{m_{F}}^{2}\right)x^{2}+....\right]$$

when *m_F_* ≠ 0, a first-order approximation is sufficient, and its energy level shift is correlated linearly with the magnetic field when a weak magnetic field is applied. When *m_F_* = 0, its energy level is insensitive to the magnetic field and is not easily disturbed by the external magnetic field. Without the first-order term, the clock transition is insensitive to the magnetic field, which will reduce the error caused by the external magnetic field disturbance. However, the second-order coefficient still exists, and affects the uncertainty of the fountain clock. From (2), we have the relationships between the Δ*F* = 0, Δ*m_F_* ≠ 0 magnetically sensitive transition frequency and the magnetic field strength as:

(3) $$\nu_{\left(3,m_{2}\right)↔\left(3,m_{1}\right)}=350,975\times 10^{4}B-13.358\times 10^{8}\left(2m_{1}-1\right)B^{2}$$

(4) $$\nu_{\left(4,m_{2}\right)↔\left(4,m_{1}\right)}=349,859\times 10^{4}B-13.358\times 10^{8}\left(2m_{1}+1\right)B^{2}$$

where *m*_2_ represents the high-energy magnetic quantum number, *m*_1_ represents the low-energy magnetic quantum number. Typically, the C-field strength of a cesium atomic fountain clock is approximately 170 nT and, according to (3) and (4), a low-frequency field with a frequency of about 600 Hz would excite the desired Δ*F* = 0, Δ*m_F_* = ±1 transition. As long as we precisely measure the Δ*F* = 0, Δ*m_F_* = ±1 transition frequency, the corresponding magnetic field strength can be calculated.

For the (0,0) transition, we have the transition frequency deduced from (2):

(5) $$\nu_{\left(3,0\right)-\left(4,0\right)}=\nu_{Cs}\left(1+\frac{1}{2}x^{2}+....\right)$$

where the second-order term is the second-order Zeeman frequency shift of the cesium atomic fountain clock ν_2nd*Zeeman*_.

The frequency difference, Δν_−1,0_, between the |*F* = 3, *m_F_* = −1〉 →|*F* = 4, *m_F_* = −1〉 and the |*F* = 3, *m_F_* = 0〉 →|*F* = 4, *m_F_* = 0〉 transitions can be measured and expressed as:

(6) $$\frac{\Delta \nu_{-1,0}}{\nu_{Cs}}=-\frac{1}{4}x-\frac{1}{32}x^{2}-....$$

Then, the second-order Zeeman frequency shift is given by:

(7) $$\frac{\nu_{2ndZ\mathrm{ee}man}}{\nu_{Cs}}=\frac{8\Delta \nu_{-1,0}^{2}}{\nu_{Cs}^{2}}\left(1-\frac{\Delta \nu_{-1,0}}{\nu_{Cs}}-....\right)$$

According to (7), second-order Zeeman frequency shift can be calculated as long as the central frequency of |*F* = 3, *m_F_* = −1〉 →|*F* = 4, *m_F_* = −1〉 magnetically sensitive Ramsey transition fringe is measured. Since the (−1,−1) magnetically sensitive Ramsey transition is 10^6^ times as sensitive to the fluctuation of C-field as (0,0) transition, as well as the C-field’s nonuniformity, the central fringe of the (−1,−1) magnetically sensitive Ramsey transition will deviate from the Rabi pedestal, it’s difficult to identify the central fringe from the large amounts of Ramsey fringes [15]. A full map of the magnetic field at all points along the atomic trajectory within and above the Ramsey cavity is acquired by using the low-frequency transition method, and the central fringe of the (−1,−1) magnetically sensitive Ramsey transition can be calculated by a numerical integration of the field “map”.

**Figure 2:**
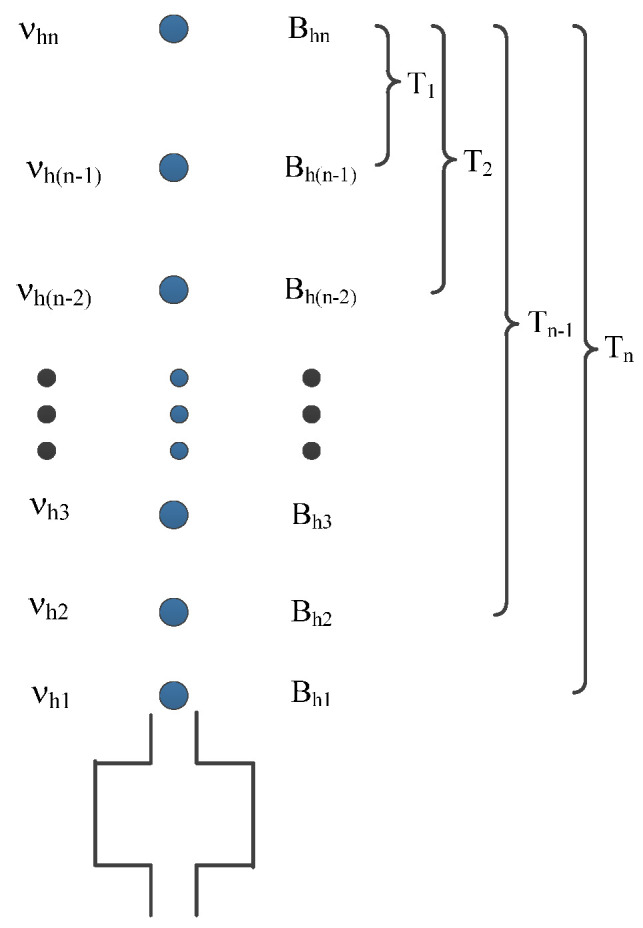
Diagram of integral solution for the central frequency of (−1,−1) Ramsey transition.

By changing the detuning frequency Δ*ν_i_* (*i* = 0, 1, 2, …, *n*) of the upward and downward triple cooling lasers, the atoms fly at different heights *h_i_* with a launching velocity *V_i_* = √3Δ*ν_i_* λ (λ = 852.35 nm). As shown in Figure 2, we suppose that the C-field strength of the first, second, …, (*n* − 1)-th, *n*-th points are B*_h_*_1_, B*_h_*_2_, *...*, B*_h_*_(*n*−1)_, B*_hn_*, and that the corresponding (−1,−1) magnetically sensitive Ramsey transition central frequencies are ν*_h_*_1_, ν*_h_*_2_, *...*, ν*_h_*_(*n*−1)_, ν*_hn_*, assuming that the time atoms spend passing from the apogee at each launching height to its bellowing first, second, …, *n*-th points are *T*_1_*, T*_2_, …, *T_n_*, respectively. By integrating the time-averaged C-field, we have the (−1,−1) magnetically sensitive Ramsey transition frequency of the first, second, …, (*n* − 1)-th, *n*-th points as follows:

(8) $$\nu_{h}{}_{1}=NB_{h1}$$

(9) $$\nu_{h2}=N\left(B_{h1}\frac{T_{2}-T_{1}}{T_{2}}+B_{h2}\frac{T_{1}}{T_{2}}\right)$$

…

(10) $$\nu_{hn}=N\left(\sum_{i=2}^{n}B_{h\left(n-i+1\right)}\frac{T_{i}-T_{i-1}}{T_{n}}+B_{hn}\frac{T_{1}}{T_{n}}\right)$$

where the coefficient *N* = 7.0083 Hz/T. In this way, we could predict the central frequency of a (−1,−1) magnetically sensitive Ramsey transition by integrating the measured C-field.

## 3. Experiment and Results

**Figure 3:**
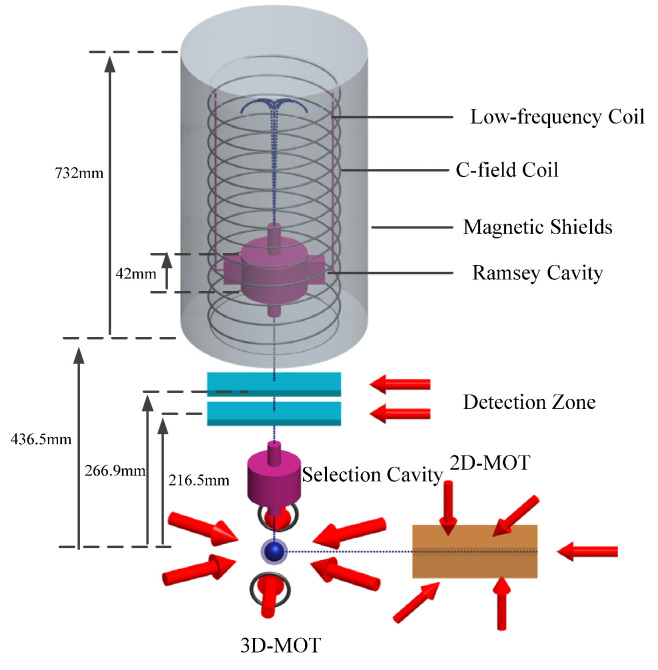
Working process of Cesium atomic fountain clock.

Figure 3 illustrates the mechanical design of the cesium atomic fountain clock. Cesium atoms are gathered in the two-dimensional magneto-optical trap (2D-MOT), which are then injected into the three-dimensional magneto-optical trap (3D-MOT). Additionally, the atoms are cooled with six laser beams in a (1,1,1) geometry, the frequencies of the vertical beams are stepped to a shifted frequency to launch the atoms up, the frequencies of the laser beams are detuned further to the red, and the intensities are decreased in order to cool the atoms more. The atoms fly upwards and enter the state-selection cavity which transfers the |4,0〉 atoms to the |3,0〉 state. Then, the atoms fly upwards and downwards pass through the microwave cavity in TE_011_ mode where the microwave excitation is performed twice, and the Ramsey transition occurs. The falling atoms eventually enters the detection zone (which has been turned on) where the relative atoms populations in the *F* = 3 and *F* = 4 hyperfine levels are measured. The measured populations of *F* = 3 and *F* = 4 levels are combined to give a normalized transition probability.

The C-field region is composed of three parts, which are the Earth’s magnetic field (about 10^5^ nT), the magnetic shielding’s residual magnetic field, and the solenoid’s magnetic field. Experimentally, a current of 1 mA is usually input to the solenoid to generate a magnetic field of appromixmately170 nT. The spatial uniformity of the C-field is subjected to the magnetic shielding’s performance, magnetic shielding demagnetization, and the winding method of the C-field solenoid. We adopt three methods to improve the uniformity of the C-field, which are four-layers high permeability cylindrical magnetic shieldings (composited of JIS C 2531 PC soft-magnetic alloy), which are assembled around the C-field solenoid, penetrating demagnetization, and a double wound C-field solenoid design [23]. A radial magnetic-field is produced with a current input to the low-frequency coil located at the solenoid, which will excite the Δ*F* = 0, Δ*m_F_* = ±1 transition. 

**Figure 4:**
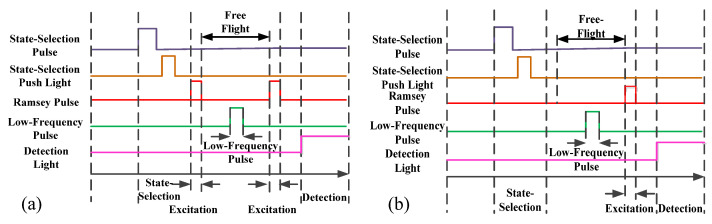
Relevant time sequence diagram of the experiment; (**a**) time sequence for the measurement of atomic flight region; (**b**) time sequence for the measurement of Ramsey cavity.

**Figure 5:**
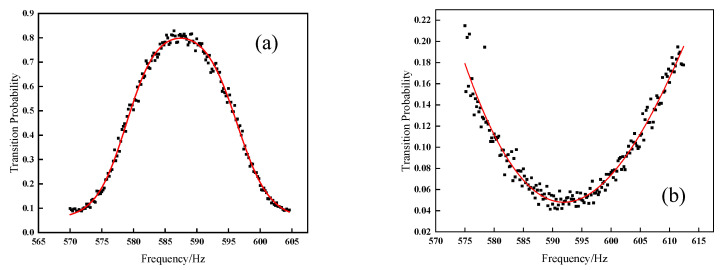
Curve of low-frequency transition; (**a**) 24 cm above the Ramsey cavity; (**b**) center of the Ramsey cavity.

According to the theoretical study in Section 2, the frequency shift of the Δ*F* = 0, Δ*m_F_* = ±1 transition is proportional to the magnetic field, which allows the measurement of the magnitude of the C-field by using the atoms as a probe at discrete locations along the atomic flight trajectory. By changing the cooling lasers‘ detuning frequency Δν*_i_*, the atoms are launched at different velocities, *V_i_*, to the desired positions *h_i_* for a magnetic field measurement. Then, those atoms in the |4,0〉 state are selected and transitioned to the |3,0〉 state in the selection cavity, and the atoms continue flying upwards to the apogee. When the measured point is above the Ramsey cavity, the related time sequence is shown in Figure 4a, the atoms in the |3,0〉 pure state enter the Ramsey cavity and realize the |*F* = 3, *m_F_* = 0〉 →|*F* = 4, *m_F_* = 0〉 Rabi transition (with the microwave field amplitude corresponding to a π pulse area), then the atoms leaving the Ramsey cavity are all excited in the *F* = 4 state. When the atoms reach to the desired height, an AC-current is input to the low-frequency coil to excite the |*F* = 4, *m_F_* = 0〉 →|*F* = 4, *m_F_* ≠ 0〉 transition. Then, atoms in *F* = 4 state fall back to the Ramsey cavity again, which transfers the remaining |4,0〉 atoms to the |3,0〉 state leaving the atoms in the |*F* = 4, *m_F_* ≠ 0〉 state unchanged. The low-frequency magnetic field is turned on shortly before the atoms fly to the apogee and last for 60 ms, the atoms move approximately 4 mm in this duration, accordingly. The atoms continue falling down to the detection zone, which gives a transition probability at the particular frequency and strength of the low-frequency magnetic field applied near the apogee. By scanning the frequency of low-frequency pulse in step 0.2 Hz, a low-frequency transition curve is obtained as shown in Figure 5a. By fitting this curve, the central frequency obtained is substituted into (4), the magnetic field strength at the particular apogee is calculated. When the measured point is inside the Ramsey cavity, the related time sequence is shown in Figure 4b. The state-selected atoms in the |3,0〉 state fly upwards and enter the Ramsey cavity first time—without microwave excitation. A low-frequency pulse is also applied when the |3,0〉 atoms near the apogee, which realizes the |*F* = 3, *m_F_* = 0〉 →|*F* = 3, *m_F_* ≠ 0〉 transition. In the same way, a transition curve is obtained by scanning the low-frequency pulse as shown in Figure 5b. Fitting this curve and substituting the central frequency into (3), the corresponding magnetic field strength inside the Ramsey cavity is measured.

**Figure 6:**
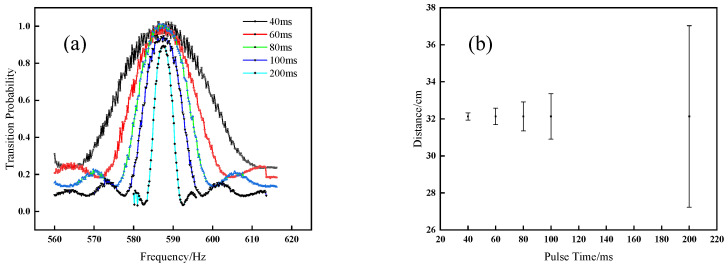
Influence of low-frequency pulse time on transition curves and atomic moving distance; (**a**) low-frequency transition curves with different low-frequency pulse time; (**b**) atomic moving distance with different low-frequency pulse time.

The purpose of measuring the magnetic field by the low-frequency transition method is to optimize the magnetic field along the atomic flight trajectory, and to thereby obtain the (1,1) or ((−1,−1)) magnetically sensitive Ramsey fringe with a high quality. The magnetic field measured by the low-frequency transition method is an averaged magnetic field in the atoms’ moving distance during the low-frequency pulse time. The low-frequency pulse time not only affects the atoms’ moving distance, but also affects the linewidth of the transition curve. Launching the atoms to an apogee of 24 cm above the Ramsey cavity, and studying the influence of low-frequency pulse time on the accuracy of magnetic field measurement by changing the low-frequency pulse time to 40 ms, 60 ms, 100 ms and 200 ms, respectively, the corresponding low-frequency transition curves are shown in Figure 6a. From Figure 6a, we can see that the longer the low-frequency pulse time is, the narrower the linewidth of the resonant curve is, which eventually means a more accurate measurement of the magnetic field. The relationship between the low-frequency pulse time and the linewidth of the transition curve satisfies the uncertainty principle of Fourier transform:

(11) $$\bar{{\left(\Delta E\right)}^{2}}\cdot \bar{{\left(\Delta t\right)}^{2}}\geq \frac{\hbar^{2}}{4}$$

where Δt is the low-frequency pulse time, and ΔE = *h*Δν,ν is the frequency of atoms. We can see that the longer the low-frequency pulse time is, the smaller change of the atomic frequency Δν is; that is, the narrower the line-width of the low-frequency transition curve is. However, with the low-frequency pulse time increasing, the corresponding atomic moving distance is longer, and the measurement of the magnetic field is more inaccurate. The influence of the low-frequency pulse time on the atomic moving distance is illustrated in Figure 6b. When the pulse time is shorter than 60 ms, the atomic moving distance is approximately 4 mm, which is close to the size of the atom cloud. When the pulse time is longer than 60 ms, the atomic moving distance is twice the size of the atom cloud, meaning that the low-frequency pulse acting on the atoms is not uniform and the accuracy of magnetic field measurement decreases. Considering the influence of the low-frequency pulse time on the atomic moving distance and the precision of finding the central frequency of the transition curve, we choose 60 ms as the pulse time.

**Figure 7:**
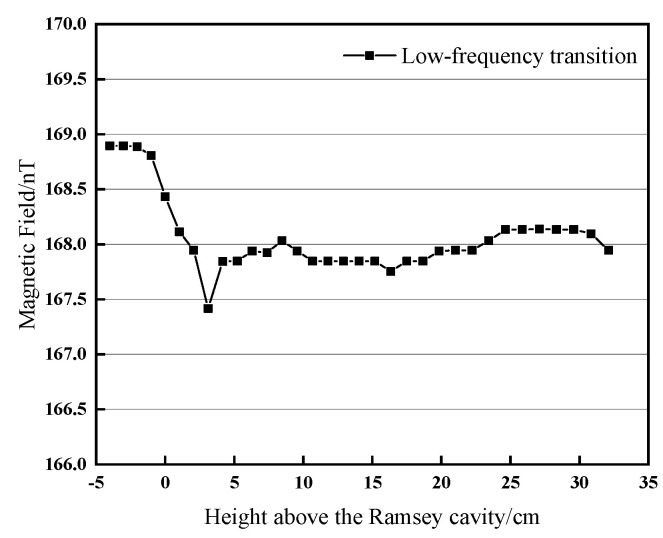
Map of the magnetic field in and above the Ramsey cavity.

Substituting the structural parameters of our cesium atomic fountain clock, we obtain a detuning frequency of Δ*ν*_0_ = 2.22 MHz, when the atoms’ launching height is just at the bottom of the Ramsey cavity. By slowly increasing the detuning frequency Δ*ν_i_* from 2.22 MHz to 2.86 MHz with a 0.02 MHz increment, and the apogee height *h_i_* rises by approximately 1 cm, and the transition curve is obtained. Repeating the entire process described in Section 3, a full map of the magnetic field inside the Ramsey cavity and along the atomic flight trajectory is finally realized. Figure 7 shows the magnetic field measured and adjusted by the low-frequency transition method, the fluctuation of the magnetic field within 32 cm of the atomic flight trajectory is less than 1 nT, the relative uniformity is 0.6%, and the fluctuation of the magnetic field inside the Ramsey cavity is less than 0.5 nT. Whether it is the manufacturing process of the Ramsey cavity or the constituent material (oxygen-free copper) of the Ramsey cavity, they all determine that the Ramsey cavity is not 100% non-magnetic. Because of the small amount of magnetic material in the Ramsey cavity, there is a nonuniformity near the upper end of the Ramsey cavity. Under such a uniform magnetic field, we can observe a symmetrical (−1,−1) or ((1,1)) magnetically sensitive Ramsey transition fringe.

**Figure 8:**
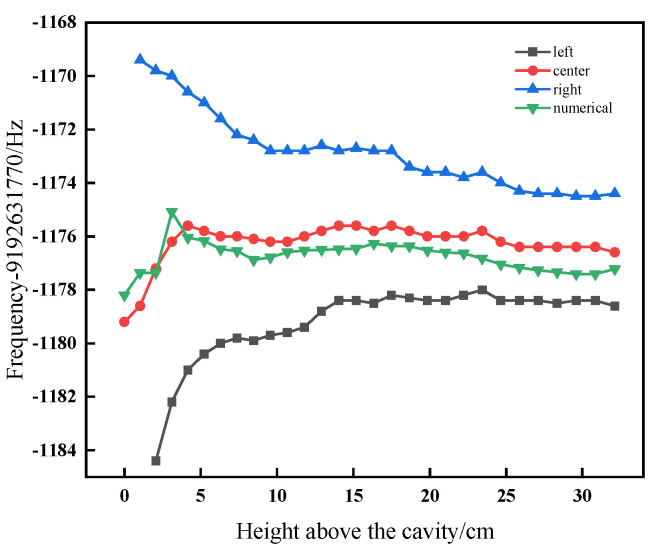
Measured three fringes and the calculated central fringe for (−1,−1) magnetically sensitive Ramsey transition as a function of apogee height above the Ramsey cavity.

We can calculate the central fringe of the (−1,−1) Ramsey transition of the desired height, by applying (10) to integrate the magnetic field distribution shown in Figure 7. Figure 8 illustrates the comparison of the central frequencies of the (−1,−1) magnetically sensitive Ramsey transition as a function of the launch height *h*, which are measured by a numerical and the Ramsey method, respectively, as the dot marked curve is obtained by the Ramsey method [24], and the green triangular marked curve is obtained by the numerical method. At the same time, we record the central frequencies of its two adjacent fringes, and obtain their variation as the square and the bule triangular marked curves shown in Figure 8.

As shown in Figure 8, the difference between the measured and the theoretically calculated central fringe is, at most, 0.3 in width. The reason for this difference is that the magnetic field measurements in two different methods are not simultaneous and the stability of the current supply causes the magnetic field distribution to change. In addition, the theoretically calculated central fringe can be used to predict the central fringe among the multi-fringes. From Figure 8, we typically measure the central frequency as *ν_h_*_32cm_ = 9,192,630,593.6 Hz. Then, we immediately input the *ν_h_*_32cm_ to the control system and lock on this central fringe and record the fluctuation of the central frequency *ν_h_* every four seconds over ten days. The result shows that the mean value of central frequency is *ν_h_*_32cm_ = 9,192,630,593.53 Hz with a variation range of *δν_h_*_32cm_ = ±0.45 Hz. Correspondingly, the frequency difference Δν_−1,0_ between the central frequency and the clock transition frequency is Δν_−1,0_ = 1176.48 ± 0.45 Hz, substituted it into (7) the second-order Zeeman frequency shift is calculated as 131.03 × 10^−15^, with the uncertainty of 0.10 × 10^−15^.

## 4. Conclusions and Discussion

Second-order Zeeman frequency shift is a frequency bias induced by C-field, which limits the accuracy improvement of cesium atomic fountain clock. A uniform and stable magnetic field is the basis of the cesium atomic fountain clock, and the measurement and adjustment of the magnetic field is crucial to reduce the second-order Zeeman frequency shift. We study the application of the low-frequency transition method in the evaluation of the second-order Zeeman frequency shift. Firstly, we deduce the formulas of magnetic field measurement and second-order Zeeman shift calculation, respectively. Secondly, a full map of magnetic field distribution of 4 cm inside the Ramsey cavity and 32 cm along the atomic trajectory is obtained by the low-frequency transition method, and the influence of the low-frequency pulse signal duration is studied. Finally, a numerical integration of the magnetic field is applied to predict the central fringe of (−1,−1) magnetically sensitive Ramsey transition, and the second-order Zeeman frequency shift is calculated by applying the central frequency determined by the Ramsey method to the Breit-Rabi formula.
